# An FT-Raman, FT-IR, and Quantum Chemical Investigation of Stanozolol and Oxandrolone

**DOI:** 10.3390/bios8010002

**Published:** 2017-12-26

**Authors:** Tibebe Lemma, Fabiano de Barros Souza, Claudio A. Tellez Soto, Airton A. Martin

**Affiliations:** 1 Faculdade de Ciencias e Technologia (FCT), Universidade Estadual Paulista (UNESP), Presidente Prudente, São Paulo 19060-900, Brazil; 2 Fisiologia e Farmacodinamica, University of Vale do Paraiba (UNIVAP), Shishima Hifumi Ave, 2911, Sao Jose dos Campos, São Paulo 12244-000, Brazil; fabiano@univap.br; 3 Biomedical Engineering Innovation Center-Biomedical Vibrational Spectroscopy Group, Universidade Brasil-Rua Carolina Fonseca, Itaquera , São Paulo 235-08230-030, Brazil; amartin@pq.cnpq.br; 4 Departamento de Física, CCN, Universidade Federal do Piauí (UFPI), Bairro Ininga, Teresina/PI 64049-550, Brazil

**Keywords:** Stanozolol, Oxandrolone, FT-Infrared, FT-Raman

## Abstract

We have studied the Fourier Transform Infrared (FT-IR) and the Fourier transform Raman (FT-Raman) spectra of stanozolol and oxandrolone, and we have performed quantum chemical calculations based on the density functional theory (DFT) with a B3LYP/6-31G (d, p) level of theory. The FT-IR and FT-Raman spectra were collected in a solid phase. The consistency between the calculated and experimental FT-IR and FT-Raman data indicates that the B3LYP/6-31G (d, p) can generate reliable geometry and related properties of the title compounds. Selected experimental bands were assigned and characterized on the basis of the scaled theoretical wavenumbers by their total energy distribution. The good agreement between the experimental and theoretical spectra allowed positive assignment of the observed vibrational absorption bands. Finally, the calculation results were applied to simulate the Raman and IR spectra of the title compounds, which show agreement with the observed spectra.

## 1. Introduction

Anabolic steroids (AAS) are synthetic compounds made up of testosterone and its derivatives, originally developed for pure medical and scientific purposes. These synthetic forms of testosterone have drawn increasing attention due to their unique ability to maximize the anabolic effect (muscle-building) while minimizing androgenic (masculinizing) side effects. At physiological concentrations, AAS plays an essential role in the biological activity of humans’ biology and other organisms. The reported compounds featured multiple functions, including stimulation of bone growth, appetite, puberty [1], muscle mass, and muscle growth [2]. The most common use of anabolic steroids is in chronic wasting conditions (debilitating diseases) such as advanced cancer and acquired immune deficiency syndrome (AIDS) [3]. Anabolic steroids also can produce numerous physiological effects including virilization [4,5], increased protein synthesis [6,7,8], muscle mass, strength, appetite, and bone growth [2]. However, despite their legitimate medical uses, anabolic steroids are misused and exploited by several individuals for a variety of reasons including athletic performance, increase muscle mass, strength, and appearance [9,10,11], which have both physiological and metabolic consequences. 

Today, anabolic steroids are controversial because of widespread misuse by several athletes to enhance physical performance and physical appearance. Improper uses and high doses of anabolic steroids show adverse side effects in both genders. These effects include cosmetic changes such as acne, baldness, gynecomastia, and testicular atrophy in men; clitoromegaly, facial hair, and lowering of the voice in women; as well as a number of high risk health factors such as elevated cholesterol (LDL increases and HDL decreases), stroke, elevated blood pressure, liver failure , cardiac arrhythmia, and infraction [12,13]. In addition, the misuse may cause a serious physiological, psychological, and social problems, which are associated with an increasing mood swings, aggression, conduct disorder, and body dysmorphic disorder [14,15,16,17].

There has been considerable interest over the past decades in the development of effective anabolic agents that can involve pharmacological interventions and can be safely used to promote anabolism, with a targeted primarily loss of metabolically active lean tissue of patients for the past several decades. Two such agents are stanozolol and oxandrolone, which have been developed as oral formulations for therapeutic purposes based on its the anabolic (muscle-building) and androgenic (masculinizing) properties. Stanozolol, usually sold under the names Winstrol (oral) and Winstrol Depot (intramuscular), was developed by Winthrop Laboratories in 1962. Oxandrolone was first synthesized by Pfizer Inc. under the trade name Anavar in 1964. 

Stanozolol (ST) is a synthetic derivative of the male sex hormone testosterone, first synthesized in 1959 [18]. It exists at room temperature as a white crystal and is practically insoluble in water. However, it is soluble in alcohol, chloroform, and dimethylformamide solvents. Stanozolol has been used on human patients and animals for various conditions. In humans, it has demonstrated its success in treating anemia and hereditary angioedema [3]. Veterinarians may prescribe the drug to improve muscle growth and the production of red blood cells, to increase bone density, and to stimulate the appetite for weak or debilitated animals [19]. However, it is also misused [20] by some athletes, especially bodybuilders, to lose fat while preserving muscle mass. Stanozolol is among the anabolic steroids most used as ergogenic agents, and its use is banned in athletic competitions under the rules of the International Association of Athletics Federations (IAAF) [21]. The drug received great publicity after Ben Johnson tested positive for stanozolol at the Seoul Olympics in 1988 [22].

Oxandrolone (Ox) is also part of the class of anabolic steroids, an analog of testosterone, that have been used clinically in adults to treat muscle wasting in AIDS patients, wasting syndrome, as well as in burned children to improve protein synthesis and lean muscle mass [23]. It is designed for use in adults and children, and, when administered correctly it has been shown to decrease post-traumatic catabolism and improve muscle protein synthesis in burned patients [24]. Nevertheless, it has not always been used for the purely medical purposes. Due to its minimal side effects with regard to liver toxicity, Ox was sought after by many athletes as a performance enhancement drug because of its ability to enhance muscular strength with few side effects [25]. 

Due to their considerable importance, a deeper understanding of the chemistry and structure of these steroids is needed, in order to develop a vibrational-based, precise method that will be able us to distinguish the stanozolol and Ox molecules from other anabolic steroids. Various studies on anabolic steroids have been widely investigated by several analytical techniques [26]. However, very little work has been done on the analysis of the fundamental vibrations of stanozolol and oxandrolone. 

Vibrational spectroscopy has proven very useful for the study of the molecular basis of anabolic steroids [27,28]. Indeed, Raman spectroscopy has been shown to be a powerful experimental tool for determining subtle conformational order changes in a wide variety of biologically related materials. Although the methods have been demonstrated, the extent of scientific literature on the vibrational spectroscopy of anabolic steroids is very limited, with very few articles having been published so far. The aim of this work is to present Raman and infrared spectra, followed by an attempt to correlate the experimental vibrational peaks in the Raman and FT-IR spectra to the results of the DFT (Density Functional Theory) calculations in order to understand the nature of vibrations in the anabolic steroids’ molecules. The IR spectrum of stanozolol [29,30] has been previously reported; however, the present work is the first comprehensive analysis of these vibrational spectra with respect to quantum chemical calculations. This paper is a part of a systematic study of the vibrational spectra of anabolic steroids originating from testosterone and its synthetic analogs.

## 2. Experimental 

Raman measurements were carried out using a Bruker RFS-100/s system (Bruker, Karlsruhe, Germany). The 1064 nm line of a Nd:YAG laser was used as the excitation source in the region of (0 to 4000 cm^−1^) with the power set in the range 30–100 mW. The laser was focused on a spot with a diameter approximately 2 µm. The acquisition mode for all spectra was set to 1000 to 2000 scans. Some spectral regions of the average spectra were also analyzed by the normalized least-squares curve-fitting procedure (peak fit module of Origin 8.6 software) using multiple Voigt (Gaussian-Lorentzian mix) curves. The FT-IR spectra of stanozolol and oxandrolone powder were recorded with 2 cm^−1^ resolution using a PerkinElmer spectrometer 400, equipped with a nitrogen-cooled mercury cadmium telluride (MCT) detector. KBr was used as a beam-splitter and the spectral resolution set to 2 cm^−1^. Raw spectra were manipulated by built-in functions in the OPUS software, which performs corrections in the atmospheric water vapor and CO_2_ absorption bands, together with baseline and smoothing operations. 

### 2.1. Materials and Methods

Stanozolol (17β-hydroxy-17α-methylandrostano [3,2-c] pyrazole) (ST) of (%) and oxandrolone (17β-hydroxy-17α-methyl-2-oxa-5α-androstan-3-one) were obtained from Aldrich. All chemicals were used without further purification.

### 2.2. Computational Details

**Figure 1:**
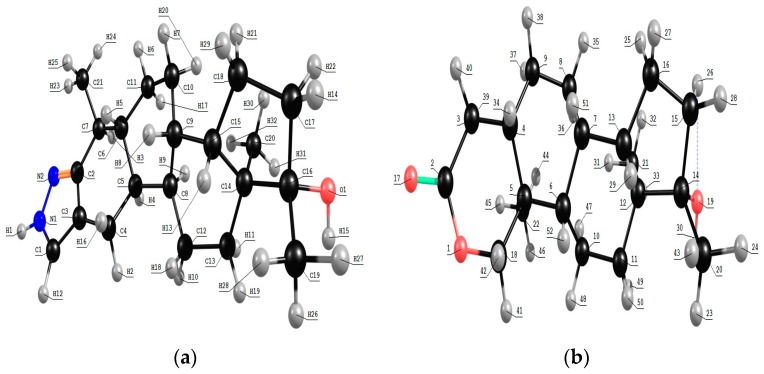
The optimized structure of (**a**) stanozolone and (**b**) oxandrolone molecules calculated with B3LYP/6-311G (d, p) theoretical level.

The molecular structure of the compounds studied were optimized (Figure 1) at the DFT level using the Lee-Yang-Parr correlation functional (B3LYP) [31] and 6-311G (d, p) [32] level of theory for the ground state of stanozolol and oxandrolone with a C1 symmetry. Raman and IR wavenumbers, as well as the band intensities, were calculated at the same DFT level using the GAUSSIAN 98W program [33]. Harmonic vibrational wavenumbers were calculated using analytical second derivatives to confirm the convergence to minimal in the potential surface. At the optimized structure of the examined species, no imaginary wavenumber modes were obtained, provided that a true minimum on the potential surface was found. The calculated and experimental values were compared using the scaling factors [34] method to offset the systematic errors caused by the evaluated wavenumbers for vibrational anharmonicity and deficiencies inherent to the computational level used. The good agreement between the experimental and theoretical spectra allowed positive assignment of the observed vibrational bands. Finally, the calculated results were applied to simulate the Raman and IR spectra of the title compound, and these showed good agreement with the observed spectra.

## 3. Results and Discussion

### 3.1. Vibrational Analysis

**Figure 2:**
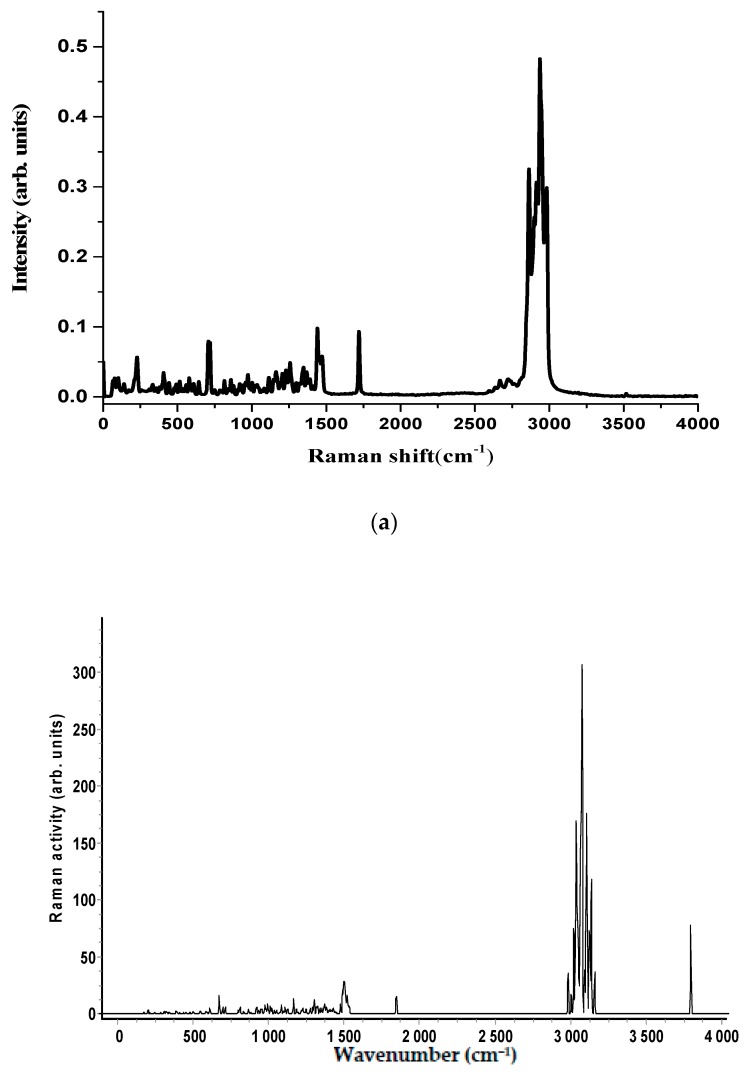
(**a**) FT-Raman spectrum of oxandrolone molecule in the wavenumber range 4000–0 cm^−1^; (**b**) The simulated Raman spectra of oxandrolone computed with a B3LYP/6-311G (d, p) level of theory. Experimental conditions: power = 30–100 mW, number of scans = 1000–2000 scans, exposure time = 10 s for each spectrum. Baselines have been subtracted for all the spectra.

**Figure 3:**
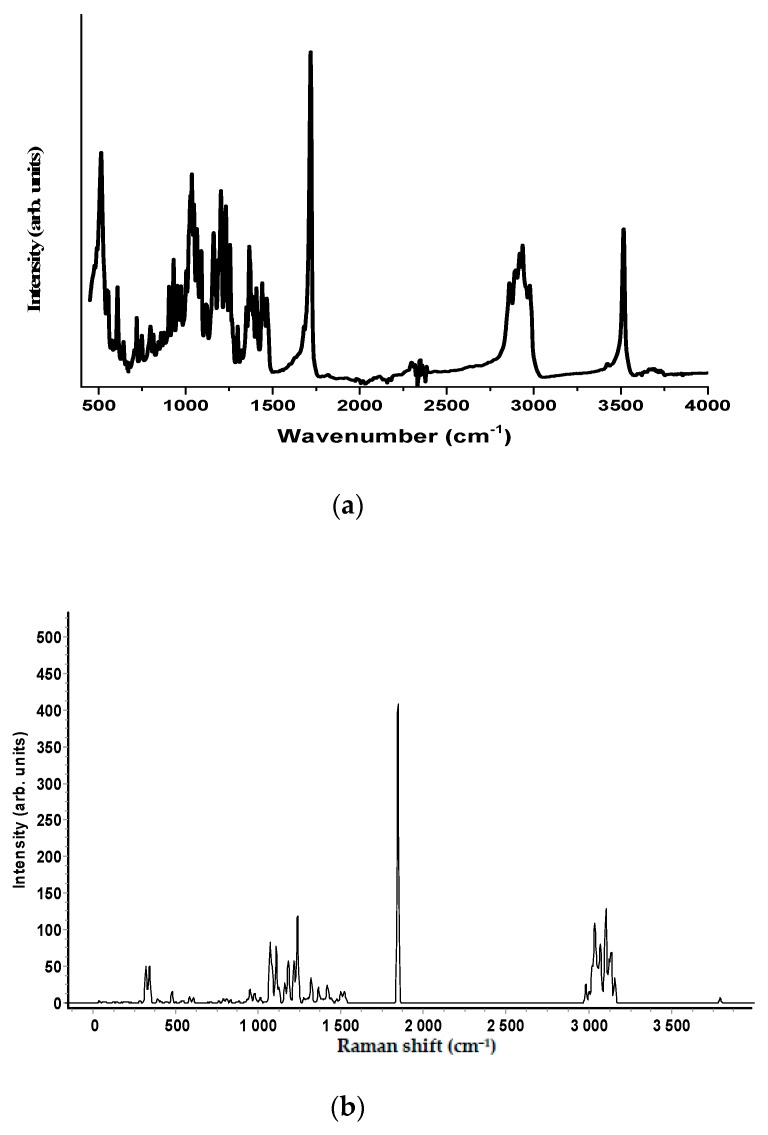
(**a**) The FT-IR spectrum of oxandrolone molecule in the wavenumber range 4000–400 cm^−1^; (**b**) The simulated FT-IR spectra of oxandrolone computed with a B3LYP/6-311G (d, p) level of theory.

**Figure 4:**
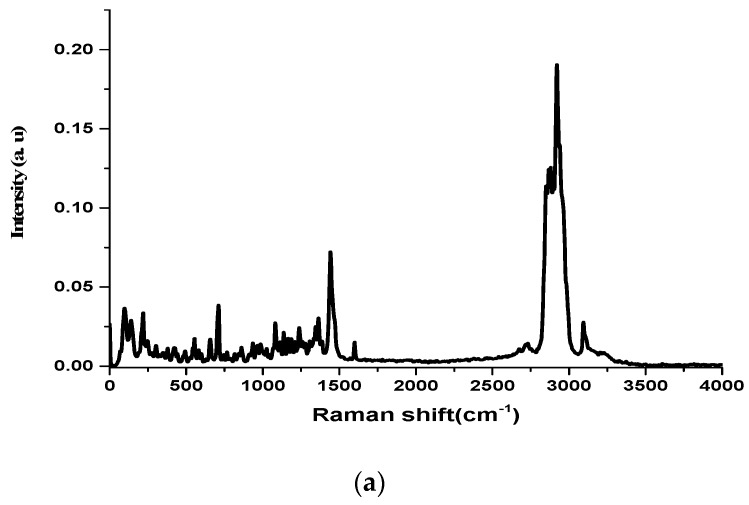

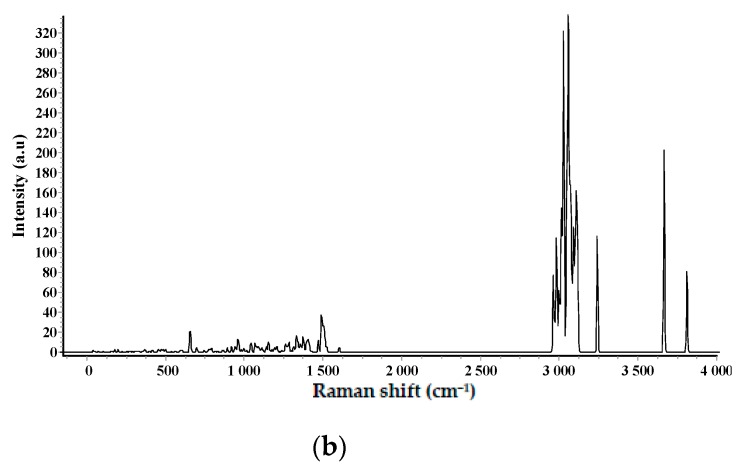
(**a**) FT-Raman spectrum of stanozolone molecule in the wavenumber range 4000–0 cm^−1^; (**b**) The simulated Raman spectrum of stanozolone computed with a B3LYP/6-311G (d, p) level of theory.

**Figure 5:**
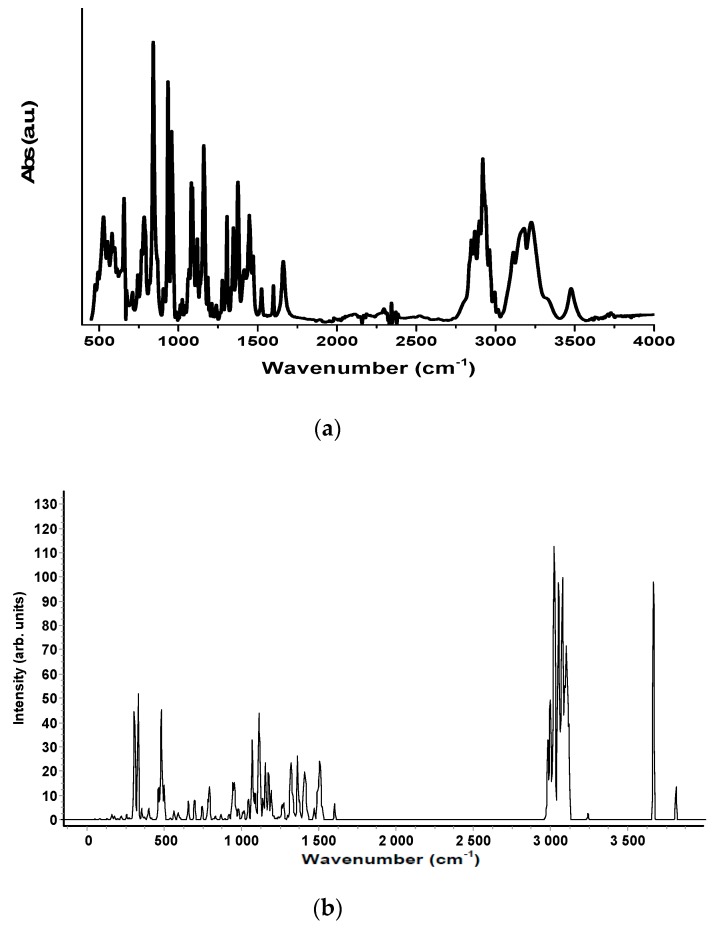
(**a**) FT-IR spectrum of stanozolone molecule in the wavenumber range 4000–400 cm^−1^; (**b**) The simulated FT-IR spectra of stanozolone computed with a B3LYP/6-311G (d, p) level of theory.

The experimental Raman and the infrared spectra of the two anabolic steroids, together with the calculated vibrational spectra, are shown in Figure 2, Figure 3, Figure 4 and Figure 5. The complete assignment of all the vibrational modes is given in Table 1 and Table 2. The spontaneous Raman scattering spectra and the infrared spectra contain an extended number of fundamental vibrational frequencies that are reasonably well predicated by the quantum chemical computation. Most of the bands in the Raman spectrum are also present in the observed IR spectrum. The caveat is that the observed relative intensities in the spectra of the solid may not agree with the calculated spectrum, which would correspond to a gas-phase spectrum, and significant differences between the calculated and observed vibrational intensities could be expected. 

The vibrational spectra analysis is performed on the basis of the characteristic vibrations of the OH, CO, and CH groups of oxandrolone and stanozolon. The oxandrolone and stanozolone group vibrations encompass the carbonyl, hydroxyl, pyrrole, and lactone groups. As can be seen from Figure 2, Figure 3 and Figure 4, and from Table 1 and Table 2, the prediction of the DFT calculations for the vibrational wavenumbers are in good agreement with the experimental values and allow for a complete assignment of the vibrational modes, with almost one-to-one peak correspondence, especially for the Raman spectrum simulations.

In the following discussion, we will focus on the assignments of intense vibrational modes that characterize oxandrolone and stanozolon. The vibrations of these groups may be divided into the stretching and deformation vibrations of hydroxyl, the stretching and deformation vibrations of CH, and the stretching vibrations of C—O.

### 3.2. Assignments of Vibrational Modes

#### 3.2.1. Oxandrolone

As the symmetry of the oxandrolone molecule is C1, all 150 vibrational modes are both infrared and Raman active. We find 72 strong or medium intensity bands in both IR and Raman spectra, yielding 55 stretching, 113 bending (including redundancies coordinates), and 24 torsion internal coordinates.

##### O—H Stretching Vibration 

The hydroxyl stretching vibrations are generally observed around 3500 cm^−1^. The broad and intense band observed in IR at 3516 cm^−1^ corresponds to the O—H stretching vibration. The shift to higher wavenumber suggests intra-and intermolecular O—H^....^O hydrogen bonding in the molecule. Near this band in the infrared spectrum, the overtone of the ν(C=O) vibrational mode at 3423 cm^−1^ (2 × 1718 = 3436 cm^−1^) appears.

##### C—H Stretching Vibrations

The C—H vibrations of both molecules are clearly observed in IR and Raman spectra between 2984–2850 cm^−1^, with strong bands as expected. In the oxandrolone molecule, there are —CH_3_, —CH­_2_, and —CH (methyne) groups. The bands assigned to the ν(CH) vibrational modes are observed in the range of wavenumbers between 2984 and 2850 cm^−1^. Not all the stretching C—H bands were observed, due to the higher overlapped band inside of the contour of the band observed in the above-indicated region. These assignments are considered to be straightforward because they belong to the characteristic groups —CH_3_ and —CH_2_ with very well defined spectra [34,35,36]. Our assignments are given in Table 1.

##### C=O Stretching

The most intense band in the infrared spectrum is assigned to the ν(C=O) vibrational mode, which was found at 1718 cm^−1^. In the Raman spectrum, this mode was found at 1720 cm^−1^. The intensities of this band in both spectra in a normalized scale are: 0.99 in the infrared spectrum and 0.19 in the Raman spectrum. The intensity ratio in both spectra agree very well with one of the selection rules of vibrational spectroscopy concerning the dipole moment (higher intensity bands in the infrared spectra), and with the polarizability (Raman spectra). 

##### CH Deformation Vibrations

Bending normal modes are found in the spectral region between 1679 and 1065 cm^−1^, including the δ(HCH)(CH_3_), δ(HCH)(CH_2_­), and δ(CCH)(methine groups). By the natural structural conformation of the oxandrolone molecule, all these bending normal modes must be coupled by different internal bending coordinates, which are present in the δ(HCH)(CH_3_), δ(HCH)(CH_2_­), and δ(CCH)(methine groups). The assignments are given in Table 1. Rocking vibrations of these chemical groups are found in the spectral region between 973 and 796 cm^−1^. Due to the aliphatic nature of the compounds, these bending frequencies according to Bellamy [25] are considered also as characteristic bands and they are localized at C—CH_3_ between 1450 ± 20 cm^−1^ for the asymmetric vibrations and between 1465 ± 20 cm^−1^ for the —CH_2_ groups with mean intensities. 

##### CCC Deformation Vibrations

The molecular structure of oxandrolone includes three six-member rings and one ring formed of five atoms. From these structures, we expected to find eleven δ(CCC) and one δ(COC) bending modes. The assignments of these modes can be considered as coupled, and are found in the spectral region between 898 and 515 cm^−1^. The molecular structure of oxandrolone includes three six-member rings and one ring formed of five atoms. From this structure, we expected to find eleven δ(CCC) and one δ(COC) bending modes. The assignments of these modes can be considered as coupled with different angular bending internal coordinates, having also 18 redundant coordinates in the six and five-member rings. The pyran ring also contains three redundant internal bending coordinates. The wavenumbers for these vibrations are found in the spectral region between 898 to 515 cm^−1^.

##### CC and CO Stretching Vibrations 

CC and CO stretching vibrations appears in the region between 1188 and 952 cm^−1^. All of them are present as coupled modes with different CC and CO stretching internal coordinates, and with bending internal coordinates. Table 1 (Table S1, Supplementary Material) presents the assignment for these CC and CO coupled vibrational modes.

##### Low-Frequency Vibrations: Torsional Normal Modes

Different types of torsional vibrations are present in the oxandrolone molecule: those which belong to the —CH_3_, —CH_2_, and —CH groups, and those which are present in the four rings of the geometrical structure of the molecule. These vibrational modes are found in the region between 491 and 67 cm^−1^. The vibrational assignments are present in Table 1 (Table S1, Supplementary Material).

#### 3.2.2. Stanozolol 

Molecular Formula: C_21_H_32_N_2_O. Stanozolol has 3^n−6^ = 162 normal modes. For this molecule, we can define 60 stretching, 122 bending, and 28 torsion internal coordinates, including redundancies.

##### O—H, N—H and C—H Ring Stretching Vibrations

The ν(OH) stretching vibrational mode was observed at 3474 cm^−1^ in the infrared spectrum. The ν(NH) vibrational mode has a band at 3320 cm^−1^ in the infrared spectrum. No Raman bands were found for these two vibrational modes. However, the ν(CH) of the unsaturated ring was observed at 3014 cm^−1^ in the infrared spectrum. 

##### CH Stretching

In the stanozolol molecule, there are —CH_3_, —CH_2_, and —CH (methyne) groups. The bands assigned to the ν(CH) vibrational modes are observed in the range of wavenumbers between 2996–2838 cm^−1^. Not all the stretching C—H bands were observed due to the higher overlapped band inside the contour of the band observed in the above indicate region. Our assignment is given in Table 2 (Table S1, Supplementary Material).

##### C=C and C=N Stretching

The ν(C=C) and ν(C=N) vibrational modes were observed only in the infrared spectrum at 1525 and 1415 cm^−1^, respectively. Both normal modes are coupled. The first one can be described as ν(C=C) + ν(C=N), and the second mode as: δ(C=NH) + ν(CC), according to the DFT calculations. In our opinion, this DFT assignment is doubtful, because the observed spectral range for C=N stretching is 1660 to 1480 cm^−1^, and for the C=C stretching, the skeletal in-plane vibrations are in the spectral range between 1600 to 1450 cm^−1^. One of the reasons for the coupled vibration is due to a minor difference (0.5 units) in electronegativity [37].

##### CH Deformation Vibrations

Bending normal modes are found in the spectral region between 1471 and 1088 cm^−1^, including the δ(HCH) (CH_3_), δ(HCH)(CH_2_), and δ(CCH) (methine groups). For the natural structural conformation of the oxandrolone molecule, all these bending normal modes must be coupled by different internal bending coordinates, which are present in the δ(HCH)(CH_3_), δ(HCH)(CH_2_­), and δ(CCH) (methine groups). The assignments are given in Table 1. Rocking vibrations of these chemical groups are found in the spectral region between 1062 and 706 cm^−1^.

##### CCC Deformation Vibrations

The molecular structure of stanozolol includes three six-member rings and two rings formed of five atoms. From these structures, we expected to find 11 δ(CCC) and one δ(NNC) bending modes. These vibrational modes are strongly coupled, in which rocking internal coordinates of —CH_3_ and —CH­_2_ groups also participate. Approximated assignments of these modes are given in Table 2. Coupled vibrations and redundant coordinates inside the rings were done in the oxandrolone molecule, which presents analogous structural elements.

##### N—N, C—C, and N—C Stretching

These vibrational modes occur within the range 1142 to 1026 cm^−1^ [25]. All of them are coupled vibrational modes, as we can see in the assignments given in Table 2 (Table S1, Supplementary Material).

##### Low-frequency Vibrations: Torsional Normal Modes

As we have discussed for the oxandrolone molecule, stanozolol also has different types of torsional vibrations that are present: those which belong to the —CH_3_, —CH_2_, and —CH groups, and those which are present in the five rings of the geometrical structure of the molecule. These vibrational modes are found in the region between 591 and 71 cm^−1^ . The vibrational assignments are present in Table 2 (Table S1, Supplementary Material).

Although our calculations allow us to obtain insights into the nature of different Raman and IR modes, it should be remembered that the experimental spectra show a significantly smaller number of bands than predicted theoretically. This can be attributed to the fact that some modes have very similar energies and the corresponding bands are not resolved in the spectra observed for the two molecules.

## 4. Conclusions

In this work, the FT-IR and FT-Raman spectral measurements have been made for the stanozolol and oxandrolone molecules. The complete vibrational analysis of the title compounds was performed on the basis of DFT calculations at the B3LYP/6-31G (d, p) basis set. The consistency between the calculated and experimental FT-IR and FT-Raman data indicates that the B3LYP/6-31G (d, p) method can generate reliable geometry and related properties of the title compounds. The difference between the observed and scaled wavenumber values of most of the fundamentals is very small. This conclusion is of great importance for the possible application of vibrational spectroscopy coupled with Surface Enhanced Raman Spectroscopy (SERS) as a diagnostic tool, since it consistently shows promising techniques. Therefore, the information presented in this paper can be used to facilitate the detection of a broad range of steroids, particularly the elusive anabolic steroids. A good knowledge of steroid structural elucidation will allow us to detect and identify a number of designed steroids and assess the relative biological activity of these compounds.

## Figures and Tables

**Table 1:** Theoretical calculation (in cm^−1^) of oxandrolone calculated by DFT (B3LYP/6-311G (d, p)) and its comparison with the experimental data.

Calculated ^a^/cm^−1^	IR Intensity ^b^	Raman Activity ^c^	Calculated ^d^	Observed/(cm^−1^) IR	Observed/(cm^−1^) Raman	Assignments ^e^
3796	7.65	78.42	3649	3516 (0.44)	3516 (0.01)	ν(OH)
				3423 (0.13)		2 × 1720 = 3440
3138	20.70	53.66	3017	2984 (0.26)	2983 (0.62)	ν(CH)(CH_3_)
3107	19.47	69.24	2987	2968 (0.28)	2967 (0.48)	ν(CH)(CH_3_)
1846	429.75	18.40	1775	1718 (0.99)	1720 (0.19)	ν(C=O)
1535	2.08	6.22	1476	1679 (0.23)		δ(HCH)(CH_2_)(CH_3_)
1529	3.96	5.14	1470	1473 (0.23)	1472 (0.12)	δ(HCH)(CH_2_)(CH_3_)
1523	3.02	6.27	1464	1466 (0.28)		δ(HCH)(CH_2_)(CH_3_)
1516	3.70	4.99	1457		1457 (0.12)	δ(HCH)(CH_2_)(CH_3_)
1508	1.60	10.79	1450	1450 (0.28)		δ(HCH)(CH_2_)(CH_3_)
1371	0.73	10.08	1318	1319 (0.15)	1319 (0.04)	δ(HCH)(CH_2_)(CH_3_)(CH) + δ(COH)
1352	0.68	0.70	1300	1299 (0.19)	1298 (0.05)	δ(HCH)(CH_2_) + δ(COH)
1252	2.63	2.38	1204	1203 (0.49)	1204 (0.07)	δ(HCH)(CH_2_) + ν(CC)
1238	120.76	1.17	1190	1188 (0.31)	1190 (0.04)	ν(CO) + δ(HCH)(CH_2_)
1217	57.05	2.99	1170	1171 (0.29)	1169 (0.06)	ν(CO) + ν(CC) + δ(HCH)(CH2)
1196	7.38	1.38	1150	1161 (0.37)	1162 (0.07)	ν(CC) + ρ(CH3)
1186	24.48	4.24	1140	1144 (0.17)	1146 (0.05)	δ(HCH)(CH2) + ν(CC)
1161	19.45	1.59	1116	1117 (0.19)	1115 (0.06)	δ(HCH)(CH2) + ν(CC) + δ(COH)
1128	17.42	1.78	1084	1091 (0.31)	1092 (0.02)	ν(CC) + δ(HCH)(CH2) + δ(COH)
1125	3.89	3.04	1081	1083 (0.24)	1084 (0.03)	ν(CC) + δ(HCH)(CH2)
1111	76.56	5.73	1068	1065 (0.37)	1167 (0.02)	δ(COH) + ν(CC) +δ(HCH)(CH2)
1086	30.68	6.06	1044	1047 (0.43)	1046 (0.03)	ν(CC) + δ(CCC)
1013	7.81	7.22	974	973 (0.25)	973 (0.06)	ρ(CH_2_)
1000	0.97	2.18	961		959 (0.04)	ν(CC)
994	1.822	9.12	956	952 (0.25)	954 (0.04)	ν(CC) + ρ(CH_2_)
976	3.17	5.60	938	931 (0.32)	931 (0.04)	ρ(CH_2_) + ρ(CH_3_)
958	9.10	3.68	921		919 (0.04)	ρ(CH_3_) + ν(CO)
951	14.78	4.27	914	905 (0.26)	907 (0.02)	ρ(CH_2_) + ρ(CH_3_)
868	1.32	4.00	834	838 (0.16)	839 (0.02)	δ(CCC)
835	4.53	2.21	803	815 (0.19)	815 (0.05)	ρ(CH_2_) + ρ(CH_3_) + δ(CCC)
813	5.80	5.32	782	798 (0.21)	800 (0.02)	δ(CCC)
807	0.96	2.18	776	786 (0.17)	785 (0.02)	ρ(CH_2_)
790	5.90	0.25	759	749 (0.19)	750 (0.02)	δ(COC) + ρ(CH_2_)
763	2.99	0.29	733	719 (0.23)	719 (0.16)	δ(CCC)
715	1.55	6.57	687	706 (0.16)	707 (0.16)	δ(CCC)
698	0.75	5.52	671		664 (0.00)	δ(CCC)
673	0.17	16.39	647	644 (0.17)	644 (0.05)	δ(CCC)
612	0.38	3.59	588	609 (0.30)		δ(CCC)
609	6.93	1.94	585	590 (0.16)		ρ(CH_3_) + δ(CCC)
594	0.87	1.29	571	578 (0.15)	579 (0.06)	δ(CCC)
587	8.05	1.53	564	556 (0.22)	558 (0.04)	δ(CCC)
550	2.43	2.14	529	549 (0.20)	551 (0.02)	δ(CCC)
543	0.27	0.58	522	530 (0.20)	536 (0.02)	δ(CCC)
537	2.41	0.40	516	515 (0.46)	516 (0.05)	δ(CCC)
503	1.83	1.57	484	491 (0.17)	492 (0.04)	τ(CCCC)

^a^ B3LYP:6-31G (d, p) calculation. Observed and calculated values in cm^−1^; ^b^ Units are Km·mol^−1^; ^c^ Units are Å^4^ (amu)^−1^; ^d^ Scaled *ab initio* calculations with factors of 0.9613 used for all modes; ^e^ υ, Stretching; δ, bending; ρ, rocking; τ, torsion; wagg, wagging; twist, twisting; sciss, scissoring.

**Table 2:** Wavenumbers (cm^−1^) of observed and calculated bands in the infrared and Raman spectra of stanozolol.

Calculated ^a^/cm^−1^	IR Intensity ^b^	Raman Activity ^c^	Calculated ^d^	Observed/(cm^−1^) IR	Observed/(cm^−1^) Raman	Assignments ^e^
3813	13.66	82.33	3665	3474 (0.19)		ν(OH)
3667	103.89	205.73	3525	3320 (0.19)		N—H
3243	2.53	116.64	3118	3014 (0.22)		ν(CH)
3114	19.63	78.08	2994	2996 (0.28)	2992 (0.19)	ν(CH)(CH_3_)
3048	14.58	40.76	2930	2929 (0.60)		ν(CH)(CH_2_) + ν(CH)(CH_3_)
3045	26.92	38.01	2927	2920 (0.72)	2921 (0.95)	ν(CH)(CH_2_) + ν(CH)(CH_3_)
3021	77.88	28.09	2904	2900 (0.50)	2900 (0.58)	ν(CH)(CH_2_)
2985	21.41	28.08	2870	2869 (0.45)	2867 (0.63)	ν(CH)(CH_2_)
2983	16.08	90.09	2868		2851 (0.59)	ν(CH)(CH_2_)
2973	3.90	27.18	2858	2848 (0.41)	2849 0.60	ν(CH)(CH_2_)
1603	6.74	4.86	1541	1525 (0.12)		ν(C=N) + ν(C=C)
1524	5.51	5.13	1465	1471 (0.24)	1469 (0.16)	δ(HCH)(CH_2_)sciss.
1501	4.08	5.81	1443	1442 (0.28)	1443 (0.38)	δ(HCH)(CH_2_)(CH_3_)sciss.
1470	5.18	12.11	1413	1415 (0.20)		δ(N=NH) + ν(CC)
1427	3.79	0.78	1372	1381 (0.31)	1387 (0.08)	δ(HCH)(CH_3_)
1415	12.20	2.16	1361	1375 (0.49 )		δ(HCH)(CH_3_) + δ(HCH)(CH_2_)twist.
1412	2.53	3.31	1357		1365 (0.16)	δ(HCH)wagg. + ν(C=N) + ν(C—C)
1400	0.71	6.08	1346	1347 (0.33)	1346 (0.13)	δ(CCH) + δ(HCH)(CH­_2_)twist.
1394	0.51	7.33	1340	1335 (0.14)	1333 (0.10)	δ(CCH) + δ(HCH)(CH­_2_)wagg.
1314	14.14	3.80	1264	1264 (0.06)		δ(HCH)(CH_2_)wagg. + δ(COH)
1260	5.62	5.00	1211	1210 (0.09)	1210 (0.08)	δ(HCH)(CH_2_)twist, wagg. + ν(NN) + ν(CN)
1191	11.38	4.05	1145	1142 (0.12)		ν(NN) +ν (CN) + δ(HCH)(CH_2_)twist.
1138	3.25	2.35	1094	1088 (0.51)		δ(CCC)ring + δ(HCH)(CH_2_)twist.
1120	26.80	1.31	1076	1081 (0.54)	1083 (0.14)	ν(CC) + ρ(CH_3_) + ρ(CH_2_)
1111	42.41	4.18	1068	1068 (0.25)	1068 (0.06)	δ(COH) + ρ(CH_2_) + δ(CCC)ring
1098	2.61	3.43	1055	1062 (0.22)		δ(COH) + ρ(CH_2_)
1088	8.58	4.35	1046	1042 (0.12)	1042 (0.04)	ν(CC) + ρ(CH_3_) + δ(CCC)ring
1076	9.69	3.01	1034	1026 (0.12)	1025 (0.05)	ν(CC) + ρ(CH_3_)
1068	32.86	9.10	1026	1012 (0.10)	1013 (0.04)	δ(CNN) + δ(CCH/NCH)
1040	3.15	4.03	1000	1001 (0.05)	1001 (0.05)	ρ(CH_3_) + ρ(CH_2_)
1006	2.94	0.42	967	986 (0.06)	986 (0.07)	ν(CC) + δ(CCC)
996	0.35	3.99	957	957 (0.68)	963 (0.07)	ν(CC) + δ(CCC)
980	4.77	2.62	942	934 (0.84)	936 (0.08)	ρ(CH_2_) + δ(CCC)
954	14.40	1.37	917	913 (0.12)	912 (0.04)	ρ(CH_3_) + δ(NNC)
919	2.34	5.63	883	868 (0.22)	868 (0.04)	ρ(CH_3_)
893	0.31	4.39	858		862 (0.07)	ρ(CH_3_)
868	2.25	0.94	834	841 (1.00)	846 (0.05)	ρ(CH_3_) + ν(CC)
862	0.31	1.41	829	816 (0.15)	817 (0.04)	ρ(CH_2_) + δ(CCC)
791	8.84	2.17	761	744 (0.16)	746 (0.04)	ρ(CH) ↑ + ρ(CH_2_)
604	0.33	1.10	581	591 (0.10)		τ(CCCC)
598	0.99	1.82	575	582 (0.16)	582 (0.06)	τ(CCCC)
538	0.77	0.42	517	527 (0.22)	526 (0.03)	τ(CCCC)
499	15.25	2.34	480	493 (0.08)	493 (0.05)	τ(CCCC) + ρ(NH) ↑

^a^ B3LYP:6-31G (d, p) calculation. Observed and calculated values in cm^−1^; ^b^ Units are Km·mol^−1^; ^c^ Units are Å^4^ (amu)^−1^; ^d^ Scaled *ab initio* calculations with factors of 0.9613 used for all modes; ^e^ υ, Stretching; δ, bending; ρ, rocking; τ, torsion; wagg, wagging; twist, twisting; sciss, scissoring.

## Supplementary Materials

The following are available online at www.mdpi.com/2079-6374/8/1/2/s1, Table S1: Theoretical calculation (in cm^−1^) of oxandrolone calculated by DFT (B3LYP/6-311+G (d, p)) and their comparison with the experimental data, Table S2: Wavenumbers (cm^−1^) of observed and calculated bands in the infrared and Raman spectra of stanozolol.

## References

[1] Basaria S., Wahlstrom J., Dobs A. (2001). Anabolic-androgenic steroid therapy in the treatment of chronic diseases. J. Clin. Endocrinol. Metab..

[2] Shahidi N. (2001). A review of the chemistry, biological action, and clinical applications of anabolic-androgenic steroids. Clin. Ther..

[3] Lok S., Tasgin E., Yalcin H. (2012). Morphometric effects of the combined usage of anabolic androgenic steroids on humerus. Energy Educ. Sci. Technol. Part B Soc. Educ. Stud..

[4] Barragry T. (1974). Anabolic steroids. Irish. Vet. J..

[5] Ziegler T., Leader L., Jonas C. (1997). Adjunctive therapies in nutritional support. Nutrition.

[6] Gruber A.J., Pope H.G.J. (2000). Psychiatric and medical effects of anabolic-androgenic steroid use in women. Psychother. Psychosom..

[7] Malarkey W., Strauss R., Leizman D., Liggett M., Demers L. (1991). Endocrine effects in female weight lifters who self-administer testosterone and anabolic steroids. Am. J. Obstet. Gynecol..

[8] Kanayama G., Boynes M., Hudson J., Field A. (2007). Anabolic steroid abuse among teenage girls: An illusory problem?. Drug Alcohol Depend..

[9] Carson J.A., Lee W.J., McClung J., Hand G.A. (2002). Steroid receptor concentration in aged rat hindlimb muscle: effect of anabolic steroid administration. J. Appl. Physiol..

[10] Strawford A., Barbieri T., Loan M., Parks E., Catlin D., Barton N., Neese R., Christiansen M., King J., Hellerstein M. (1999). Resistance exercise and supraphysiologic androgen therapy in eugonadal men with HIV-related weight loss. JAMA.

[11] Bates P., Chew L., Millward D. (1987). Effects of the anabolic steroid stanozolol on growth and protein metabolism in the rat. J. Endocrinol..

[12] Oberlander J.G., Porter D.M., Penatti C.A.A., Henderson L.P. (2012). Anabolic Androgenic Steroid Abuse: Multiple Mechanisms of Regulation of GABAergic Synapses in Neuroendocrine Control Regions of the Rodent Forebrain. J. Neuroendocrinol..

[13] Brower K., Blow F., Young J.P., Hill E.M. (1991). Symptoms and correlates of anabolic-androgenic steroid dependence. Brit. J. Addict..

[14] Oda S., El-Ashmawy I.M. (2012). Adverse effects of the anabolic steroid, boldenone undecylenate, on reproductive functions of male rabbits. Int. J. Exp. Pathol..

[15] Brower K.J., Catlin D.H., Blow F.C., Eliopulos G.A., Beresford T.P. (1991). Clinical asessment and urine testing for anabolic-androgenic steroid abuse and dependence. Am. J. Drug Alcohol Abus..

[16] Creagh T.M., Rubin A., Evans D.J. (1988). Hepatic tumours induced by anabolic steroids in an athlete. J. Clin. Pathol..

[17] Friedl K., Lin G., Erinoff L. (1990). Reappraisal of the health risks associated with the use of high doses of oral and injectable androgenic steroids. Anabolic Steroid Abuse.

[18] Tonya D., Hoagland M.F. (2011). The Use of Anabolic Androgenic Steroids and Polypharmacy: A Review of the Literature. Drug Alcohol Depend..

[19] Clinton R.O., Manson A.J., Stonner F.W. (1959). Steroidal [3,2-c] pyrazoles. J. AM. Chem. Soc..

[20] Poelmans S., Wasch K., De Brabander H., Van De Wiele M., Courtheyn D. (2002). Analytical possibilities for the detection of stanozolol and its metabolites. Anal. Chim. Acta.

[21] Beckett A., Cowan D. (1979). Misuse of drugs in sport. Brit. J. Sports Med..

[22] Tucci P., Morgese M.G., Colaianna M., Zotti M., Schiavone S., Cuomo V., Trabace L. (2012). Neurochemical consequence of steroid abuse: Stanozolol-induced monoaminergic changes. Steroids.

[23] Hoberman J. (2005). Testosterone Dreams: Rejuvenation, Aphrodisia, Doping.

[24] Hart D., Wolf S., Ramzy P., Chinkes D.L., Beauford R.B., Ferrando A.A., Wolfe R.R., Herndon D.N. (2001). Anabolic effects of oxandrolone after severe burn. Ann. Surg..

[25] Wolf S.E., Thomas S.J., Dasu M.R., Ferrando A.A., Chinkes D.L., Wolfe R.R., Herndon D.N. (2003). Improved Net Protein Balance, Lean Mass, and Gene Expression Changes With Oxandrolone Treatment in the Severely Burned. Ann. Surg..

[26] Freriks K., Sas T.C.J., Traas M.A.F., Netea-Maier R.T., Heijer M.D., Hermus A.R.M.M., Wit J.M., Velden J.A.E.M.V.A.V.D., Otten B.J., Keizer-Schrama S.M.P.F.D.M. (2013). Long-term effects of previous oxandrolone treatment in adult women with Turner syndrome. Eur. J. Endocrinol..

[27] Deshmukh N.I., Zachar G., Petróczi A., Székely A.D., Barker J., Naughton D.P. (2012). Determination of stanozolol and 3′-hydroxystanozolol in rat hair, urine and serum using liquid chromatography tandem mass spectrometry. Chem. Cent. J..

[28] Ward R., Shackleton C., Lawson A. (1975). Gas chromatographic-mass spectrometric methods for the detection and identification of anabolic steroid drugs. Br. J. Sports Med..

[29] Minaeva V., Minaev B., Hovorun D. (2008). Vibrational spectra of the steroid hormones, estradiol and estriol, calculated by density functional theory. The role of low-frequency vibrations. Ukr. Biokhim. Zh.

[30] Chiong D.M., Consuegrarodriguez E., Almirall J.R. (1992). The Analysis and Identification of Steroids. J. Forensic Sci..

[31] Becke A.D. (1988). Correlation energy of an inhomogeneous electron gas: A coordinate-space model. J. Chem. Phys..

[32] Mclean A.D., Chandler G.S. (1980). Contracted Gaussian basis sets for molecular calculations. I. Second row atoms, Z=11–18. J. Chem. Phys..

[33] Frisch M.J., Trucks G.W., Schlegel H.B., Scuseria G.E., Robb M.A., Cheeseman J.R., Montgomery J.A., Vreven T., Kudin K.N. Gaussian, Inc., Wallingford CT, 2004. Gaussian 03, Revision C.02. http://gaussian.com/g03citation/.

[34] Anthony P.S., Leo R. (1996). Harmonic Vibrational Frequencies: An Evaluation of Hartree-Fock, Møller-Plesset, Quadratic Configuration Interaction, Density Functional Theory, and Semiempirical Scale Factors. J. Phys. Chem..

[35] Bellamy L.J. (1966). The infra-red Spectra of Complex Molecules.

[36] Sverdolov L.M., Kovner M.A., Krainov E.P. (1974). Vibrational Spectra of Polyatomic Molecules.

[37] Allred A.L. (1961). Electronegativity values from thermochemical data. J. Inorg. Nucl. Chem..

