# Supplementary material for: An FT-Raman, FT-IR, and Quantum Chemical Investigation of Stanozolol and Oxandrolone
Source: Biosensors (Basel). 2017 Dec 26;8(1):2. doi: 10.3390/bios8010002 (PMC5872050; doi:10.3390/bios8010002)
Supplement: Supplementary file 1 [file biosensors-08-00002-s001.pdf]

Supplementary Materials: FT-Raman, FT-IR and Quantum Chemical Investigation of Stanozolol and Oxandrolone

Tibebe Lemma, Fabiano de Barros Souza, Claudio A Tellez Soto and Airton A. Martin

Table 1. Theoretical calculation (in cm^{-1}) of oxandrolone calculated by DFT (B3LYP/6-311+G (d, p)) and their comparison with the experimental data.

Calculate [a]/cm^{-1}	IR Intensity [b]	Raman Activity [c]	Calculated [d]	Observed/(cm^{-1}) IR	Observed/(cm^{-1}) Raman	Assignments [e]
3796	7.65	78.42	3649	3516 (0.44)	3516 (0.01)	ν(OH)
				3423 (0.13)		2 × 1782 = 3436
3158	34.97	39.80	3036			ν(CH)(CH_3)
3138	31.35	46.82	3017			ν(CH)(CH_3)
3138	20.70	53.66	3017	2984 (0.26)	2983 (0.62)	ν(CH)(CH_3)
3135	18.52	48.33	3014			ν(CH)(CH_3)
3126	28.52	70.20	3005			ν(CH)(CH_2)
3121	38.20	61.34	3000	2977 (0.30)		ν(CH)(CH_2)
3107	32.86	78.91	2987			ν(CH)(CH_2)
3107	19.47	69.24	2987	2968 (0.28)	2967 (0.48)	ν(CH)(CH_3)
3103	39.03	50.28	2983			ν(CH)(CH_2)
3102	33.61	35.33	2982			ν(CH)(CH_2)
3100	22.36	37.14	2980	2956 (0.30)		ν(CH)(CH_2)
3092	27.83	38.95	2972			ν(CH)(CH_2)(CH_3)
3076	20.84	281.95	2957			ν(CH)(CH_2)(CH_3)
3072	10.28	65.28	2953			ν(CH)(CH_2)(CH_3)
3071	35.01	23.45	2952			ν(CH)(CH_2)(CH_3)
3069	16.36	55.41	2950			ν(CH)(CH_2)
3066	8.42	144.72	2947			ν(CH)(CH_2)(CH_3)
3062	17.54	22.40	2944		2945 (0.87)	ν(CH)(CH_2)(CH_3)
3056	28.22	24.16	2938	2937 (0.40)	2936 (0.99)	ν(CH)(CH_2)(CH_3)
3050	16.46	43.24	2932			ν(CH)(CH_2)(CH_3)
3045	41.26	94.36	2927			ν(CH)(CH_2)(CH_3)
3038	19.07	110.93	2920	2919 (0.38)		ν(CH)(CH_2)(CH_3)
3037	39.72	43.19	2919			ν(CH)(CH_2)
3033	53.60	117.66	2916		2913 (0.64)	ν(CH)(CH_2)
3021	29.07	64.04	2904	2897 (0.34)	2898 (0.53)	ν(CH)(CH_2)
3019	23.29	33.20	2902	2890 (0.34)		ν(CH)
3003	16.35	20.88	2887	2867 (0.31)		ν(CH)
2983	2.44	4.20	2868	2860 (0.31)	2863 (0.67)	ν(CH)
2983	24.77	36.47	2868	2850 (0.25)	2848 (0.28)	ν(CH)
1846	429.75	18.40	1775	1718 (0.99)	1720 (0.19)	ν(C=O)
1535	2.08	6.22	1476	1679 (0.23)		δ(HCH)(CH_2)(CH_3)
1529	3.96	5.14	1470	1473 (0.23)	1472 (0.12)	δ(HCH)(CH_2)(CH_3)
1523	3.02	6.27	1464	1466 (0.28)		δ(HCH)(CH_2)(CH_3)
1523	7.79	8.98	1464			δ(HCH)(CH_2)(CH_3)
1518	3.48	5.55	1459			δ(HCH)(CH_2)(CH_3)
1516	3.70	4.99	1457		1457 (0.12)	δ(HCH)(CH_2)(CH_3)
1511	0.25	6.33	1453			δ(HCH)(CH_2)(CH_3)
1508	1.60	10.79	1450	1450 (0.28)		δ(HCH)(CH_2)(CH_3)
1505	2.77	16.09	1447			δ(HCH)(CH_2)(CH_3)
1503	3.57	7.15	1445			δ(HCH)(CH_2)(CH_3)
1501	6.87	7.16	1443			δ(HCH)(CH_2)(CH_3)
1498	2.80	22.69	1440	1440 (0.31)	1441 (0.20)	δ(HCH)(CH_2)(CH_3)
1490	1.65	22.93	1432	1435 (0.25)		δ(HCH)(CH_2)(CH_3)

1477	5.59	8.77	1420	1406 (0.29)	1412 (0.03)	δ(HCH)(CH_2)(CH_3)
1449	3.67	1.41	1393	1391 (0.24)	1390 (0.08)	δ(HCH)(CH_2)(CH_3)
1439	5.63	2.00	1383	1382 (0.28)		δ(HCH)(CH_2)(CH)
1429	4.19	4.71	1374	1371 (0.35)	1369 (0.07)	δ(HCH)(CH_2)(CH_3)
1423	11.74	1.82	1368	1366 (0.40)		δ(HCH)(CH_2)(CH_3)
1417	16.80	3.12	1362			δ(HCH)(CH_2)(CH_3)(CH)
1408	0.74	4.29	1354			δ(HCH)(CH_2)(CH_3)(CH)
1403	4.58	0.52	1349	1350 (0.24)		δ(HCH)(CH_2)(CH_3)(CH)
1399	0.87	2.75	1345		1347 (0.09)	δ(HCH)(CH_2)(CH_3)(CH)
1390	4.83	1.17	1336		1339 (0.08)	δ(HCH)(CH_2)(CH_3)(CH)
1384	0.31	5.06	1330			δ(HCH)(CH_2)(CH_3)(CH)
1379	5.14	1.85	1326			δ(HCH)(CH_2)(CH_3)(CH)
1371	0.73	10.08	1318	1319 (0.15)	1319 (0.04)	δ(HCH)(CH_2)(CH_3)(CH) + δ(COH)
1366	19.33	3.97	1313			δ(COH) + δ(HCH)(CH_2)
1362	2.82	0.99	1309			δ(HCH)(CH_2)(CH_3)(CH) + δ(COH)
1352	0.68	0.70	1300	1299 (0.19)	1298 (0.05)	δ(HCH)(CH_2) + δ(COH)
1340	1.33	5.11	1288			δ(HCH)(CH_2)(CH) + δ(COH)
1327	3.04	5.09	1276	1274 (0.416)		δ(HCH)(CH_2) + δ(COH)
1324	21.46	2.79	1273			δ(HCH)(CH_2)+ δ(COH)
1318	14.55	5.62	1267	1267 (0.20)		δ(HCH)(CH_2)
1317	5.58	1.42	1266		1264 (0.06)	δ(HCH)(CH_2)
1303	6.08	12.45	1253	1255 (0.37)	1257 (0.10)	δ(HCH)(CH_2)(CH)
1293	4.74	5.92	1243	1250 (0.29)		δ(HCH)(CH_2)
1278	5.86	3.35	1229	1231 (0.46)	1229 (0.08)	δ(HCH)(CH_2) + ν(CC)
1277	1.89	1.97	1228	1224 (0.45)		δ(HCH)(CH_2) + ν(CC)
1252	2.63	2.38	1204	1203 (0.49)	1204 (0.07)	δ(HCH)(CH_2) + ν(CC)
1247	14.80	3.20	1199			δ(HCH)(CH_2) + ν(CC)
1238	120.76	1.17	1190	1188 (0.31)	1190 (0.04)	ν(CO) + δ(HCH)(CH_2)
1229	3.47	2.76	1181			ν(CC) + δ(HCH)(CH2)
1225	12.89	3.78	1178			ν(CO) + ν(CC) + δ(HCH)(CH2)
1217	57.05	2.99	1170	1171 (0.29)	1169 (0.06)	ν(CO) + ν(CC) + δ(HCH)(CH2)
1196	7.38	1.38	1150	1161 (0.37)	1162 (0.07)	ν(CC) + ρ(CH3)
1186	24.48	4.24	1140	1144 (0.17)	1146 (0.05)	δ(HCH)(CH2) + ν(CC)
1182	37.3	1.40	1136			δ(HCH)(CH2) + ν(CC)
1166	11.32	12.70	1121	1122 (0.18)		δ(HCH)(CH2) + ν(CC) + δ(COH)
1161	19.45	1.59	1116	1117 (0.19)	1115 (0.06)	δ(HCH)(CH2) + ν(CC) + δ(COH)
1128	17.42	1.78	1084	1091 (0.31)	1092 (0.02)	ν(CC) + δ(HCH)(CH2) + δ(COH)
1125	3.89	3.04	1081	1083 (0.24)	1084 (0.03)	ν(CC) + δ(HCH)(CH2)
1111	76.56	5.73	1068	1065 (0.37)	1167 (0.02)	δ(COH) + ν(CC) + δ(HCH)(CH2)
1100	4.48	1.94	1057			ν(CC) + δ(CCC)
1097	2.49	0.99	1055			ν(CC) + δ(CCC)
1086	30.68	6.06	1044	1047 (0.43)	1046 (0.03)	ν(CC) + δ(CCC)
1083	21.90	2.82	1041	1036 (0.51)	1036 (0.04)	ν(CC) + δ(CCC)
1073	80.87	1.22	1031	1027 (0.46)		ν(CO) + δ(CCC)
1055	1.83	3.03	1014	1021 (0.37)	1021 (0.03)	ν(CC) + δ(CCC)
1040	1.60	3.43	1000	1004 (0.28)	1002 (0.04)	ν(CC) + δ(CCC)
1023	1.28	5.06	983		988 (0.03)	ν(CC) + δ(CCC)
1013	7.81	7.22	974	973 (0.25)	973 (0.06)	ρ(CH_2)
1000	0.97	2.18	961		959 (0.04)	ν(CC)
994	1.822	9.12	956	952 (0.25)	954 (0.04)	ν(CC) + ρ(CH_2)

985	3.31	2.60	947			$\rho(CH_2) + \rho(CH_3)$
979	9.44	3.32	941			$\rho(CH_2) + \rho(CH_3)$
976	3.17	5.60	938	931 (0.32)	931 (0.04)	$\rho(CH_2) + \rho(CH_3)$
958	9.10	3.68	921		919 (0.04)	$\rho(CH_3) + \nu(CO)$
951	14.78	4.27	914	905 (0.26)	907 (0.02)	$\rho(CH_2) + \rho(CH_3)$
935	5.81	2.59	899			$\rho(CH_2) + \rho(CH_3)$
922	1.54	5.34	886	876 (0.17)	877 (0.04)	$\rho(CH_2) + \rho(CH_3)$
917	0.70	3.52	882	859 (0.18)	859 (0.02)	$\rho(CH_2) + \rho(CH_3)$
886	2.94	1.65	852	845 (0.17)		$\rho(CH_2) + \rho(CH_3)$
868	1.32	4.00	834	838 (0.16)	839 (0.02)	$\delta(CCC)$
835	4.53	2.21	803	815 (0.19)	815 (0.05)	$\rho(CH_2) + \rho(CH_3) + \delta(CCC)$
813	5.80	5.32	782	798 (0.21)	800 (0.02)	$\delta(CCC)$
807	0.96	2.18	776	786 (0.17)	785 (0.02)	$\rho(CH_2)$
799	1.76	2.70	768			$\rho(CH_2)$
790	5.90	0.25	759	749 (0.19)	750 (0.02)	$\delta(COC) + \rho(CH_2)$
763	2.99	0.29	733	719 (0.23)	719 (0.16)	$\delta(CCC)$
715	1.55	6.57	687	706 (0.16)	707 (0.16)	$\delta(CCC)$
698	0.75	5.52	671		664 (0.00)	$\delta(CCC)$
673	0.17	16.39	647	644 (0.17)	644 (0.05)	$\delta(CCC)$
612	0.38	3.59	588	609 (0.30)		$\delta(CCC)$
609	6.93	1.94	585	590 (0.16)		$\rho(CH_3) + \delta(CCC)$
594	0.87	1.29	571	578 (0.15)	579 (0.06)	$\delta(CCC)$
587	8.05	1.53	564	556 (0.22)	558 (0.04)	$\delta(CCC)$
550	2.43	2.14	529	549 (0.20)	551 (0.02)	$\delta(CCC)$
543	0.27	0.58	522	530 (0.20)	536 (0.02)	$\delta(CCC)$
537	2.41	0.40	516	515 (0.46)	516 (0.05)	$\delta(CCC)$
503	1.83	1.57	484	491 (0.17)	492 (0.04)	$\tau(CCCC)$
480	15.75	1.64	461	477 (0.13)	478 (0.03)	$\tau(CCCC)$
454	1.25	0.90	436		443 (0.04)	$\tau(CCCC)$
439	1.30	0.86	422		413 (0.05)	$\tau(CCCC)$
411	2.88	0.77	395		407 (0.07)	$\tau(CCCC)$
396	4.17	0.85	381		398 (0.04)	$\tau(CCCC)$
390	2.68	1.98	375		378 (0.03)	$\tau(CCCC)$
387	0.23	0.75	372		355 (0.03)	$\tau(CCCC)$
347	27.07	0.47	334		333 (0.04)	$\tau(CCOH)$
342	32.75	0.71	329			$\tau(CCCC)$
330	6.66	0.98	317			$\tau(CCCC)$
323	23.93	1.30	311		311 (0.02)	$\tau(CCCC)$
320	27.69	1.45	308			$\tau(CCOH)$
309	0.30	2.10	297		299 (0.02)	$\tau(CH_3) + \tau(CH_2)$
287	3.26	0.55	276			$\tau(CH_3) + \tau(CH_2)$
280	0.06	0.32	269		271 (0.02)	$\tau(CH_3)$
253	0.56	0.40	243		258 (0.02)	$\tau(CH_2)$
246	0.28	0.52	236			$\tau(CH_3)$
233	0.82	0.22	224		229 (0.12)	$\tau(CH_3)$
216	0.92	1.09	208		214 (0.05)	$\tau(O{=}COC)$
191	1.53	0.27	184			$\tau(COCC) + \tau(CH_3)$
179	0.17	0.22	172			$\tau(CH_3) + \tau(CH_2)$
174	0.64	0.74	167			$\tau(CCCO)$
147	1.63	0.15	141		141 (0.04)	$\tau(CCCC) + \tau(CH_3)$
127	1.59	0.08	122		102 (0.05)	$\tau(CCCC)$
80	0.31	0.09	77		94 (0.04)	$\tau(CCCC)$
76	1.82	0.28	73		78 (0.054)	$\tau(CCCC)$
60	0.99	0.50	58		67 (0.05)	$\tau(CCCC)$
41	2.89	0.24	39			$\tau(CCCC)$

[a] B3LYP:6-31G(d, p) calculation. Observed and calculated values in cm^{-1}; [b] Units are $Km{\cdot}mol^{-1}$; [c] Units are $Å^4$ $(amu)^{-1}$; [d] Scaled *ab initio* calculations with factors of 0.9613 used for all modes; [e] υ, Stretching; δ, bending; ϱ, rocking; τ, torsion; wagg, wagging; twist, twisting; sciss, scissoring.

Table 2. Wavenumbers (cm^{-1}) of observed and calculated bands in the infrared and Raman spectra of stanozolol.

Calculated [a] /cm^{-1}	IR Intensity [b]	Raman Activity [c]	Calculated [d]	Observed/(cm^{-1}) IR	Observed/(cm^{-1}) Raman	Assignments [e]
3813	13.66	82.33	3665	3474 (0.19)		ν(OH)
3667	103.89	205.73	3525	3320 (0.19)		N—H
				3226 (0.47)	3225 (0.05)	
				3180 (0.45)		
				3157 (0.44)		
					3132 (006)	
				3113 (0.39)		
					3094 (0.14)	
				3040 (0.19)		1525 × 2 = 3050
3243	2.53	116.64	3118	3014 (0.22)		ν(CH)ϕ
3121	34.07	40.96	3000			ν(CH)(CH_3)
3114	19.63	78.08	2994	2996 (0.28)	2992 (0.19)	ν(CH)(CH_3)
3110	23.10	73.03	2990			ν(CH)(CH_3)
3106	29.09	70.01	2986	2984 (0.26)	2983 (0.28)	ν(CH)(CH_3)
3101	54.54	60.34	2981		2979 (0.28)	ν(CH)(CH_2)
3093	42.33	93.08	2974			ν(CH)(CH_3)
3088	25.60	63.43	2969	2970 (0.33)		ν(CH)(CH_3)
3079	64.62	43.66	2960	2961 (0.42)		ν(CH)(CH_2)
3077	23.34	52.74	2958		2957 (0.56)	ν(CH)(CH_2)
3072	46.12	106.82	2954			ν(CH)(CH_2)
3067	23.42	105.33	2948			ν(CH)(CH_2)
3059	28.70	270.28	2941		2939 (0.73)	ν(CH)(CH_2)
3055	63.29	115.26	2936	2938 (0.56)		ν(CH)(CH_2)
3051	30.15	78.73	2933			ν(CH)(CH_2) + ν(CH)(CH_3)

3048	14.58	40.76	2930	2929 (0.60)		ν(CH)(CH_2) + ν(CH)(CH_3)
3045	26.92	38.01	2927	2920 (0.72)	2921 (0.95)	ν(CH)(CH_2) + ν(CH)(CH_3)
3029	20.44	69.36	2912			ν(CH)(CH_3)
3028	22.05	229.92	2911			ν(CH)(CH_2) + ν(CH)(CH_3)
3027	46.27	19.76	2910			ν(CH)(CH_2) + ν(CH)(CH_3)
3024	6.06	8.20	2907			ν(CH)(CH_2) + ν(CH)(CH_3)
3021	77.88	28.09	2904	2900 (0.50)	2900 (0.58)	ν(CH)(CH_2)
3015	24.33	136.68	2898			ν(CH)(CH_2)
3007	2.89	10.53	2891			ν(CH)(CH_2)
3004	9.75	22.35	2888			ν(CH)(CH_2)
2998	49.14	54.55	2882		2880 (0.65)	ν(CH)(CH_2)
2985	21.41	28.08	2870	2869 (0.45)	2867 (0.63)	ν(CH)(CH_2)
2983	16.08	90.09	2868		2851 (0.59)	ν(CH)(CH_2)
2973	3.90	27.18	2858	2848 (0.41)	2849 0.60	ν(CH)(CH_2)
2963	0.31	79.76	2848	2838 (0.38)		ν(CH)(CH_2)
1603	6.74	4.86	1541	1525 (0.12)		ν(C=N) + ν(C=C)
1524	5.51	5.13	1465	1471 (0.24)	1469 (0.16)	δ(HCH)(CH_2)sciss.
1514	3.97	4.48	1456			δ(HCH)(CH_2)(CH_3)sciss.
1513	1.30	3.30	1454			δ(HCH)(CH_2)(CH_3)sciss.
1509	9.34	3.32	1451	1452 (0.33)		δ(HCH)(CH_2)(CH_3)sciss.
1509	8.34	5.03	1451			δ(HCH)(CH_2)(CH_3)sciss.
1504	7.75	13.23	1446	1448 (0.37)		δ(HCH)(CH_2)(CH_3)sciss.
1502	3.27	4.72	1444			δ(HCH)(CH_2)(CH_3)sciss.
1501	4.08	5.81	1443	1442 (0.28)	1443 (0.38)	δ(HCH)(CH_2)(CH_3)sciss.
1497	3.35	9.41	1439			δ(HCH)(CH_2)sciss. (CH_3)twist.
1496	4.08	2.65	1438			δ(HCH)(CH_2)sciss. (CH_3)twist.
1492	1.15	9.13	1435			δ(HCH)(CH_2)sciss. + δ(CNH)
1490	4.76	14.52	1432	1423 (0.17)		δ(HCH)(CH_2)sciss. (CH_3)twist

1489	0.83	9.96	1432			δ(HCH)(CH_2)sciss. + δ(CNH)
1488	3.72	3.36	1431			δ(HCH)(CH_2)sciss. (CH_3)twist
1470	5.18	12.11	1413	1415 (0.20)		δ(N=NH) + ν(CC)
1427	3.79	0.78	1372	1381 (0.31)	1387 (0.08)	δ(HCH)(CH_3)
1415	12.20	2.16	1361	1375 (0.49)		δ(HCH)(CH_3) + δ(HCH)(CH_2)twist.
1412	2.53	3.31	1357		1365 (0.16)	δ(HCH)wagg. + ν(C=N) + ν(C—C)
1408	10.27	8.05	1353			δ(HCH)(CH_2)(CH_3)wagg. + ν(C=N)
1405	8.62	1.69	1351			δ(HCH)(CH_3)wagg. + δ(CCH)
1400	0.71	6.08	1346	1347 (0.33)	1346 (0.13)	δ(CCH) + δ(HCH)(CH_2)twist.
1400	5.29	2.88	1345			δ(CCH) + δ(HCH)(CH_2)twist.
1394	0.51	7.33	1340	1335 (0.14)	1333 (0.10)	δ(CCH) + δ(HCH)(CH_2)wagg.
1383	1.56	2.46	1329			δ(CCH) + δ(HCH)(CH_2)wagg.
1377	3.04	3.32	1323			δ(CCH) + δ(HCH)(CH_2)twist.
1373	5.54	11.78	1320	1315 (0.08)		δ(CCH) + δ(HCH)(CH_2)wagg.
1372	0.73	1.45	1318			δ(CCH) + δ(HCH)(CH_2)twist.
1362	27.93	3.09	1309		1308 (0.08)	δ(COH) + δ(HCH)(CH_2)twist.
1358	0.10	3.17	1305	1306 (0.38)		δ(HCH)(CH_2)(CH_3)wagg.
1354	1.54	5.14	1301			δ(HCH)(CH_2)(CCH)wagg.
1345	1.35	1.01	1293	1297 (0.13)		δ(HCH)(CH_2)(CCH)wagg.
1343	0.31	3.27	1291			δ(HCH)(CH_2)(CCH)wagg.
1340	1.25	6.84	1288			δ(HCH)(CH_2)(CCH)twist., wagg.
1331	9.03	15.66	1280	1277 (0.16)	1279 (0.08)	δ(HCH)(CH_2)(CCH)twist.
1332	21.19	1.60	1271		1268 (0.08)	δ(COH) + δ(HCH)(CH_2)wagg.
1314	14.14	3.80	1264	1264 (0.06)		δ(HCH)(CH_2)wagg. + δ(COH)
1312	1.87	1.38	1261		1259 (0.09)	δ(HCH)(CH_2)wagg.
1299	1.74	1.52	1249			δ(HCH)(CH_2)wagg.
1283	0.18	10.00	1234	1239 (0.08)	1239 (0.13)	δ(HCH)(CH_2)twist.
1275	3.23	3.02	1225	1234 (0.07)		δ(HCH)(CH_2)wagg. + δ(COH)

1270	4.96	2.30	1221	1223 (0.05)		δ(HCH)(CH_2)wagg.
1264	0.74	4.62	1215			δ(HCH)(CH_2)twist. + ν(NN) + ν(CN)
1260	5.62	5.00	1211	1210 (0.09)	1210 (0.08)	δ(HCH)(CH_2)twist., wagg. + ν(NN) + ν(CN)
1249	1.19	1.04	1200	1197 (0.08)		ρ(CH_3)+ ρ(CH_2)
1238	1.00	1.39	1190	1186 (0.19)	1188 (0.10)	δ(HCH)(CH_2)twist.
1224	0.57	0.68	1176	1165 (0.50)	1167 (0.09)	ν(NN) + ν(CN) + δ(HCH)(CH_2)twist.
1208	1.36	6.21	1161	1160 (0.63)		ν(NN) + δ(HCH)(CH_2)wagg.
1197	2.92	2.02	1151			δ(HCH)(CH_2)wagg. + δ(HCH)(CH_2)twist.+ ν(CC)
1191	11.38	4.05	1145	1142 (0.12)		ν(NN) +ν (CN) + δ(HCH)(CH_2)twist.
1176	21.03	2.67	1130	1136 (0.16)	1138 (0.12)	δ(HNN) + ν(NN) + δ(HCH)(CH_2)twist.
1164	3.28	1.55	1119	1119 (0.32)		δ(HNN) + ν(NN) +ν(CC) + ρ(CH_3)
1155	18.58	2.11	1110		1115 (0.08)	δ(HNN) + ν(NN) + ν(CC) + δ(HCH)(CH_2)twist.
1152	7.58	8.41	1108			ν(CC) + δ(HCH)(CH_2)wagg.
1140	5.96	3.38	1096	1098 (0.25)		ν(NN) + δ(HCH)(CH_2)twist.
1138	3.25	2.35	1094	1088 (0.51)		δ(CCC)ring + δ(HCH)(CH_2)twist.
1120	26.80	1.31	1076	1081 (0.54)	1083 (0.14)	ν(CC) + ρ(CH_3) + ρ(CH_2)
1111	42.41	4.18	1068	1068 (0.25)	1068 (0.06)	δ(COH) + ρ(CH_2) + δ(CCC)ring
1098	2.61	3.43	1055	1062 (0.22)		δ(COH) + ρ(CH_2)
1089	3.18	0.96	1047			ρ(CH_3) + ρ(CH_2)
1088	8.58	4.35	1046	1042 (0.12)	1042 (0.04)	ν(CC) + ρ(CH_3) + δ(CCC)ring
1080	1.42	2.74	1038			ν(CC) + ρ(CH_3)
1076	9.69	3.01	1034	1026 (0.12)	1025 (0.05)	ν(CC) + ρ(CH_3)
1068	32.86	9.10	1026	1012 (0.10)	1013 (0.04)	δ(CNN) + δ(CCH/NCH)
1046	0.90	0.92	1006			ρ(CH_3) + δ(CCC)
1044	5.71	5.98	1004			ρ(CH_3) + ρ(CH_2)

1040	3.15	4.03	1000	1001 (0.05)	1001 (0.05)	ρ(CH_3) + ρ(CH_2)
1016	3.81	1.85	976	990 (0.06)		ρ(CH_3) + ρ(CH_2)
1006	2.94	0.42	967	986 (0.06)	986 (0.07)	ν(CC) + δ(CCC)
996	0.35	3.99	957	957 (0.68)	963 (0.07)	ν(CC) + δ(CCC)
980	4.77	2.62	942	934 (0.84)	936 (0.08)	ρ(CH_2) + δ(CCC)
967	1.22	0.89	930			ρ(CH_2)
964	2.68	5.21	927			δ(NNC) + δ(NCH) + ρ(CH_2)
960	1.61	9.87	922			δ(NNC) + δ(NCH) + ρ(CH_2)
954	14.40	1.37	917	913 (0.12)	912 (0.04)	ρ(CH_3) + δ(NNC)
944	14.23	4.98	907	905 (0.12)		ρ(CH_3)
937	4.89	1.93	901			ρ(CH_3) + δ(CCC)
919	2.34	5.63	883	868 (0.22)	868 (0.04)	ρ(CH_3)
893	0.31	4.39	858		862 (0.07)	ρ(CH_3)
868	2.25	0.94	834	841 (1.00)	846 (0.05)	ρ(CH_3) + ν(CC)
862	0.31	1.41	829	816 (0.15)	817 (0.04)	ρ(CH_2) + δ(CCC)
830	1.29	0.89	798	792 (0.30)		ρ(CH)↑ +ρ(CH_3)
815	0.33	1.09	783	784 (0.35)		ρ(CH_2)
794	5.22	1.77	763	768 (0.24)	768 (0.05)	ρ(CH) ↑ +ρ(CH_2)
791	8.84	2.17	761	744 (0.16)	746 (0.04)	ρ(CH) ↑ +ρ(CH_2)
782	7.97	3.00	751	712 (0.07)	712 (0.20)	ν(CC) + ρ(CH)↑
773	0.34	2.54	743		706 (0.17)	ρ(CH_2)
745	5.75	1.42	716	673 (0.04)		τ(CCCN) + ρ(NCC ↑)
699	2.62	2.66	672	665 (0.09)	657 (0.10)	δ(CCC) + δ(CCN)
695	7.04	2.55	668	640 (0.06)		δ(CCC)
659	3.52	12.51	633	629 (0.05)	631 (0.02)	ρ(NH) ↑
654	5.69	14.44	629	598 (0.11)	600 (0.04)	ρ(NH) ↑ + δ(CCN)
604	0.33	1.10	581	591 (0.10)		τ(CCCC)
598	0.99	1.82	575	582 (0.16)	582 (0.06)	τ(CCCC)

589	2.96	0.76	566	560 (0.11)		τ(CCCC)
563	3.85	0.83	541	554 (0.14)	555 (0.09)	τ(CCCC)
538	0.77	0.42	517	527 (0.22)	526 (0.03)	τ(CCCC)
499	15.25	2.34	480	493 (0.08)	493 (0.05)	τ(CCCC) + ρ(NH) ↑
487	14.44	2.29	468	484 (0.05)	481 (0.03)	τ(CCCC) + ρ(NH) ↑
479	42.24	1.08	461	477 (0.07)		ρ(NH) ↑ + τ(CCCC)
472	8.24	3.06	454	473 (0.06)		τ(CCCC) + ρ(NH)↑
461	14.07	1.21	443		443 (0.04)	τ(HOCC) + τ(CCCC)
453	0.29	2.28	435		424 (0.07)	τ(CCCC)
418	0.58	2.04	402		413 (0.05)	τ(CCCC)
401	4.33	0.43	385		380 (0.06)	τ(CH_3)
393	2.24	0.37	378			τ(CH_3) + τ(CH_2)
373	1.19	1.68	359			τ(CH_3) + τ(CH_2)
364	0.38	2.21	350		349 (0.05)	τ(CCCC) + τ(CH_2)
354	4.63	0.93	340		326 (0.04)	τ(CH_3)
330	53.64	0.86	318			τ(HOCC)
321	2.71	1.03	308		304 (0.07)	τ(CH_3) + τ(HOCC)
310	19.04	0.82	298			τ(HOCC)
305	36.16	0.47	293			τ(CH_3) + τ(CH_2) + τ(HOCC)
291	0.62	0.72	280		274 (0.05)	τ(CH_3) + τ(CH_2)
278	0.16	0.79	267			τ(CH_3) + τ(CH_2)
272	0.74	0.86	261			τ(CH_3)
255	2.31	0.55	245		248 (0.09)	τ(CH_3) + τ(CH_2)
231	0.11	0.06	222		219 (0.18)	τ(CH_3) + τ(CH_2)
223	0.54	0.16	214		212 (0.12)	τ(CH_3)
220	1.09	0.31	211			τ(CH_3)
198	0.20	2.56	191		191 (0.05)	τ(CH_2)
177	1.52	2.27	170			τ(CH_3) + τ(CH_2)

160	2.15	1.23	154		τ(CH_3)
152	0.03	0.60	146	141 (0.15)	τ(CCCC)
128	0.43	0.31	123		τ(CCCC)
104	0.09	0.50	100	97 (0.19)	τ(CCCC)
82	0.45	0.07	79		τ(CCCC)
73	0.10	0.85	70	71 (0.05)	τ(CCCC)
50	0.16	0.60	48		τ(CCCC)
39	0.01	1.42	37		τ(CCCC)

[a] B3LYP:6-31G (d, p) calculation. Observed and calculated values in cm^{-1}; [b] Units are Km mol^{-1}; [c] Units are $Å^4$ $(amu)^{-1}$; [d] Scaled *ab initio* calculations with factors of 0.9613 used for all modes; [e] υ, Stretching; δ, bending; ϱ, rocking; τ, torsion; wagg, wagging; twist, twisting; sciss, scissoring.
